# Wheat Straw-Derived N-, O-, and S-Tri-doped Porous Carbon with Ultrahigh Specific Surface Area for Lithium-Sulfur Batteries

**DOI:** 10.3390/ma11060989

**Published:** 2018-06-11

**Authors:** Feng Chen, Lulu Ma, Jiangang Ren, Mou Zhang, Xinyu Luo, Bing Li, Zhiming Song, Xiangyang Zhou

**Affiliations:** 1 School of Resource and Environment, Henan University of Engineering, No. 1, Xianghe Road, Zhengzhou 451191, China; chenfeng871588@163.com (F.C.); malulu1001@163.com (L.M.); renjiangang2005@126.com (J.R.); mouzhang1015@163.com (M.Z.); hngclb@126.com (B.L.); 2 School of Metallurgy and Environment, Central South University, Lushan South Street 932, Yuelu District, Changsha 410083, China; 17307484092@163.com

**Keywords:** wheat straw, ultrahigh specific surface area, polysulfides shuttle, tri-doped porous carbon, Li-S batteries

## Abstract

Recently, lithium-sulfur (Li-S) batteries have been greeted by a huge ovation owing to their very high theoretical specific capacity (1675 mAh·g^−1^) and theoretical energy density (2600 Wh·kg^−1^). However, the full commercialization of Li-S batteries is still hindered by dramatic capacity fading resulting from the notorious “shuttle effect” of polysulfides. Herein, we first describe the development of a facile, inexpensive, and high-producing strategy for the fabrication of N-, O-, and S-tri-doped porous carbon (NOSPC) via pyrolysis of natural wheat straw, followed by KOH activation. The as-obtained NOSPC shows characteristic features of a highly porous carbon frame, ultrahigh specific surface area (3101.8 m^2^·g^−1^), large pore volume (1.92 cm^3^·g^−1^), good electrical conductivity, and in situ nitrogen (1.36 at %), oxygen (7.43 at %), and sulfur (0.7 at %) tri-doping. The NOSPC is afterwards selected to fabricate the NOSPC-sulfur (NOSPC/S) composite for the Li-S batteries cathode material. The as-prepared NOSPC/S cathode delivers a large initial discharge capacity (1049.2 mAh·g^−1^ at 0.2 C), good cycling stability (retains a reversible capacity of 454.7 mAh·g^−1^ over 500 cycles at 1 C with a low capacity decay of 0.088% per cycle), and superior rate performance (619.2 mAh·g^−1^ at 2 C). The excellent electrochemical performance is mainly attributed to the synergistic effects of structural restriction and multidimensional chemical adsorptions for cooperatively repressing the polysulfides shuttle.

## 1. Introduction

Lithium-sulfur (Li-S) batteries have attracted a lot of fashionable attention in various technology applications, ranging from portable electronic apparatuses to electric automobiles, because of their very high theoretical specific capacity (1675 mAh·g^−1^), large nominal energy density (2600 Wh·kg^−1^), low material cost, natural abundance, and environmental benignity [1,2]. Whereas the full commercialization of Li-S batteries is still hindered by several chronic issues involving inferior electroconductivity of elemental sulfur and its solid-state discharge products (i.e., Li_2_S_2_ and Li_2_S), over 80% volumetric expansion during discharge/charge processes, and dissolution of lithium polysulfides (Li_2_S_x_, 4 ≤ x ≤ 8), along with the notorious “shuttle effect” [3,4]. These issues give rise to low sulfur utilization, inferior rate capability, anode corrosion, as well as poor coulombic efficiency and integral energy efficiency [5,6].

Over decades, intense efforts have been advanced to circumvent the hurdles outlined above, such as the fabrication of various carbon-based substrate materials [3,6,7,8,9], lithium anode modification [10], developing new electrolytes or additives [11,12], and the design of novel cell configurations [13,14]. Among these procedures, a porous carbon matrix is considered one of the most promising candidates because not merely can they markedly improve the utilization of sulfur by maintaining the electrical connection, but they can also restrain the dissolution of lithium polysulfides by their abundant narrow pores and large internal surfaces [7,15]. Unfortunately, the weak physical adsorption through van der Waals’ force between the nonpolar hydrophobic carbon-based substrates and highly polar hydrophilic Li_2_S_x_ (4 ≤ x ≤ 8) obstructs the efficient trapping of dissolved polysulfides, and thus, leads to rapid capacity degradation in the long term [3,14,15].

Recently, sole oxygen-doped, nitrogen-doped, boron-doped, and sulfur-doped porous carbons have been widely explored to suppress the shuttle effect of Li-S batteries due to their strong chemical interaction with the migrated polysulfides, except for the physical adsorption [4,16,17,18]. With the introduction of doped atoms with various electronegativity (e.g., O: 3.44, N: 3.04, B: 2.04, S: 2.58), defects and active sites can incorporate into carbon-based materials, which causes the increasing of charge densities on the adjacent C atoms, in other words, improving the electronic conductivity of carbon-based materials [19,20,21]. What is more, Zhang’s group [22] proposed that these heteroatom dopants can bind polysulfides via Li-X bonds, revealing a “lithiophilic” interaction, and thus enhancing the electrochemical performance of Li-S batteries. Of late years, both experiment results and Density Functional Theory (DFT) calculation demonstrated that co-doping of carbon-based materials with two heteroatoms was found to further enhance the chemical adsorption capabilities toward lithium polysulfides by a cooperative effect with respect to sole heteroatom-doped counterparts [23,24]. Until now, much research is available related to encapsulating sulfur with heteroatom-co-doped carbon-based materials, such as hierarchical O/N-functionalized carbon [25], B- and O-dually-doped multi-walled carbon nanotubes [21], graphene-supported N- and B-rich carbon [15], O and S dual-doped 3D interconnected hierarchical porous carbons [26], and 3D coral-like N and S co-doped mesoporous carbon [27]. As reviewed above, co-doping of two heteroatoms can bring about synergistic enhancement of the sulfur immobilization. Therefore, if triple heteroatoms are simultaneously introduced into the carbon-based materials, multidimensional chemical interactions may exist between carbon substrates and lithium polysulfides, which could further improve the cycling durability of Li-S batteries. Besides, some heteroatom ternary-doped porous carbons have been successfully applied in the fields of oxygen reduction reaction (ORR), oxygen evolution reaction (OER), and hydrogen evolution reaction (HER) [28,29,30].

With the above discussions in mind, in this study, we for the first time describe the development of an efficient, low-cost, and high-yielding strategy for the fabrication of N-, O-, and S-tri-doped porous carbon (NOSPC) via pyrolysis of wheat straw, followed by KOH activation. Wheat straw, a natural subsidiary product of wheat, is readily available, widespread, and generally cheap, which is a fascinating starting material for fabricating high value-added nano-materials, such as hierarchically porous N-rich carbon for lithium ion batteries, nano-silica, activated carbon for supercapacitors, and wheat straw carbon-matrix-wrapped sulfur composites [31,32,33,34]. The resultant NOSPC shows characteristic features of in situ tri-doped heteroatoms of nitrogen, oxygen, and sulfur, good electrical conductivity, ultrahigh specific surface area, and highly porous structure. The ultrahigh specific surface area and highly porous structure not only can ensure uniform dispersion of elemental sulfur even with high mass loading, thereby enhancing the sulfur utilization, but also can provide enormous physical adsorption sites for polysulfides [1,35]. The ternary N/O/S dopants in the carbon substrates can further enhance the polysulfides-anchoring capability via multidimensional chemisorptions [16,27,36]. Taking advantage of the synergistic effects of structural restriction and multidimensional chemical adsorptions for cooperatively repressing the polysulfides shuttle, the as-obtained NOSPC-sulfur (NOSPC/S) composite delivers prominently enhanced electrochemical properties involving large initial discharge capacity, superior cycling life, and good rate performance.

## 2. Materials and Methods

### 2.1. Materials Preparation

The N-, O-, and S-tri-doped porous carbon (NOSPC) was produced via directly pyrolysis of wheat straw followed by KOH activation. In detail, the wheat straw was first cut into small segments with shears (about 3 cm), washed by deionized water and afterwards dried at 60 °C for 12 h. Twenty g of wheat straw was put in the plumbago crucible and pyrolyzed in a sealed box-type furnace at 400 °C for 90 min under N_2_ atmosphere with a heating-up velocity of 5 °C·min^−1^. Then, the activation was carried out by calcinating a mixture of the above obtained product and KOH with a weight ratio of 1:5 at 800 °C for 120 min in a sealed box-type furnace under N_2_ flow at a heating-up velocity of 5 °C min^−1^. After cooling to the ambient temperature spontaneously, the activated carbon was collected, neutralized with 0.5 M HCl solution, washed by DI water until a pH equaling to 7.0, and afterwards dried at 105 °C in an oven for 24 h, and the consequent samples were named as NOSPC. As a comparison, the unactivated wheat straw carbon (marked as WSC) was also produced via pyrolysis of wheat straw at 400 °C for 90 min, and followed by at 800 °C for 120 min under N_2_ atmosphere in a sealed box-type furnace except with KOH activation (the other steps are the same as that of NOSPC).

The NOSPC-sulfur (NOSPC/S) composite was fabricated via the representative melt-diffusion strategy. In detail, the above NOSPC and sulfur powder with a weight ratio of 35:65 were ground in a mortar for 30 min to ensure uniform mixing. This mixture was placed in a small glass bottle, and then transferred into 80 mL Teflon-lined stainless steel autoclave filled with Ar protection in a glove box. The stainless steel autoclave was fetched out from the glove box and put in a drying oven, finally heated at 155 °C for 12 h, and subsequently for an extra 2 h at 220 °C. As a comparison, the WSC/S composite containing the same sulfur mass ratio was also produced by the above technique.

### 2.2. Structural Characterization

The structures and morphologies of the as-prepared materials were characterized by scanning electron microscope (SEM) (JSM-6360LV, Tokyo, Japan) provided with an X-ray energy dispersive spectrometer (EDS), X-ray diffractometer (XRD, Rigaku-TTRIII, Tokyo, Japan), and Raman spectra (LabRAM Hr800, HORIBA Jobin Yvon, Tokyo, Japan). The sulfur contents in NOSPC/S and WSC/S composites were gauged by a thermogravimetric analyzer (TGA, SDTQ600, TA Instruments, New Castle, DE, USA) in a N_2_ atmosphere from indoor temperature to 800 °C with a heating-up velocity of 10 °C·min^−1^. The specific surface area, pore volume, and pore size distribution of the samples were determined by N_2_ gas adsorption at 77 K, with a self-propelled adsorption apparatus (ASAP 2020 HD88, Micromeritics, Norcross, GA, USA). To study surface chemical ingredients and function groups of the samples, X-ray photoelectron spectroscopy (XPS) tests were carried out on a K-Alpha 1063 Ultra spectrometer (Thermo Fisher Scientific, Cambridge, MA, USA). Elemental analysis was done using a EuroEA3000 (Leeman, Capitol Heights, MD, USA) analyzer.

### 2.3. Electrochemical Measurements

The cathode electrodes were prepared by mixing the carbon/sulfur composites, acetylene black, and PVDF binder with a weight ratio of 8:1:1, and the mixture was dispersed into NMP solvent to form the electrode slurry. The slurry was uniformly casted on the Al foil with a scraper blade. Then, the Al foil was dried at 50 °C overnight under the vacuum. The Al foil was chopped into round pieces with a diameter of 1 cm for use as the working electrode. The sulfur areal mass loading of the cathode electrodes was about 1.0–1.2 mg·cm^−2^. CR2025 coin cells were assembled in an Ar-filled glove box (Super 1220/750, MIKROUNA, Shanghai, China), with H_2_O and O_2_ levels below 0.1 ppm. The electrolyte was 1 mol·L^−1^ lithium bis-(trifluoromethanesulfonyl)imide (LiTFSI) in a mixture of equivalent volumes of 1,3-dioxolane (DOL) and dimethoxyethane (DME) with 0.1 mol·L^−1^ LiNO_3_ additives, and the related amount of electrolyte was 25 μL for a coil cell. The separator was a Celgard 2400 membrane and the anode was lithium foil. The galvanostatic discharge/charge tests were implemented within the voltage scope of 1.7–2.8 V versus Li^+^/Li at indoor temperature by employing a cell testing system (LAND CT-2001A, Wuhan LAND ekectronics Limited by Share, Wuhan, China). The specific capacity of the cell was based on active sulfur (1 C = 1675 mA·g^−1^). The cyclic voltammogram (CV) test was carried out at the sweep speed of 0.2 mV·S^−1^ within the voltage scope of 1.6–3.0 V by a PARSTAT 4000 electrochemistry workstation (AMETEK, San Diego, CA, USA). The electrochemical impedance spectroscopy (EIS) measurement was performed via PARSTAT 4000 electrochemistry workstation between the frequency ranges from 100 kHz to10 mHz with a 5 mV response excursion.

## 3. Results and Discussion

Wheat straw, a subsidiary product of mature wheat, has a natural multilayer structure composed of three different kinds of polymers, namely, cellulose, hemicellulose, and lignin. These polymers cross-link mutually and constitute the stem of wheat straw [37,38]. The SEM images of a single wheat straw stem are shown in Figure S1, we can see that the stem is mainly formed as coaxial circles leaving a lumen at the center (Figure S1a,b). The outer surface of the stem is a hydrophobic waxy layer to provide extra mechanical strength of the stem and inhibit the erosion of external moisture (Figure S1c). The interior of the outer surface owns many loose layers, which cross-linked each other to form a network structure (Figure S1d). Moreover, wheat straw primarily consists of C atoms, O atoms, and some other nonmetal and mineral elements [31,32]. In view of its lamellar microstructure and chemical composition, it will be easily transformed from wheat straw into highly valuable heteroatom-doped porous carbon via facile carbonization and KOH activation. The KOH activation can also adjust the specific surface area and pore volume of carbon, which is beneficial for enhancing the sulfur content and ensuring the uniform dispersion of sulfur. In addition, the raw material (i.e., wheat straw) is readily available, widespread, generally cheap, and renewable. Therefore, our strategy for the fabrication of NOSPC/S is efficient, low-cost, and high-yielding, which is suitable for the commercialized applications of Li-S batteries.

**Figure 1:**
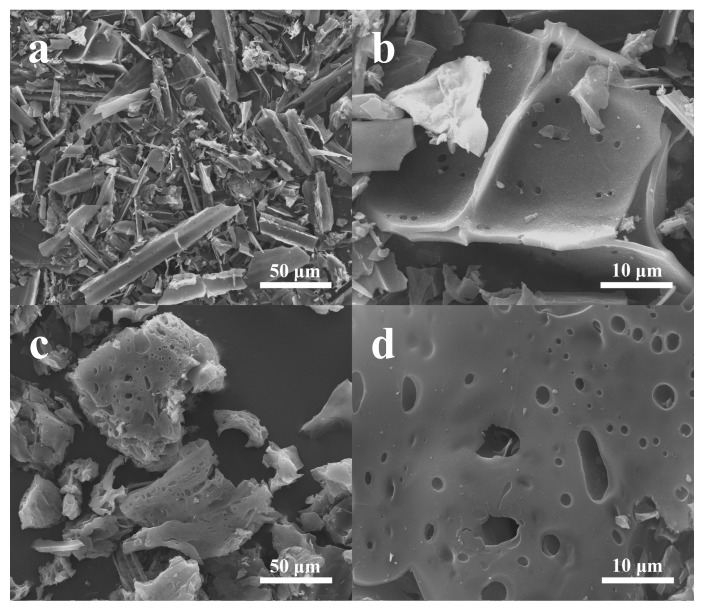
SEM images of wheat straw carbon (WSC) (**a**,**b**) and N-, O-, and S-tri-doped porous carbon (NOSPC) (**c**,**d**).

The structures and morphologies of as-prepared WSC, NOSPC, WSC/S, and NOSPC/S were first characterized by SEM. As illustrated in Figure 1a,b, the WSC displays a typical sheet structure with a diameter ranging from dozens to one hundred micrometers. Moreover, most of the WSC surface is relatively smooth except for some small voids, which maybe derive from the release of pyrolysis gases during the carbonization process [39]. Figure 1c,d exhibit the SEM images of NOSPC that did not undergo grinding; it can be clearly noticed that the KOH activation did not influence its lamellar nanostructure, but plenty of macropores are observed and mesopores have formed on the surface of NOSPC. Such a highly porous structure of NOSPC can not merely promote the rapid transport of lithium ions and electrons, but also can be beneficial for the active sulfur encapsulation [3,17].

**Figure 2:**
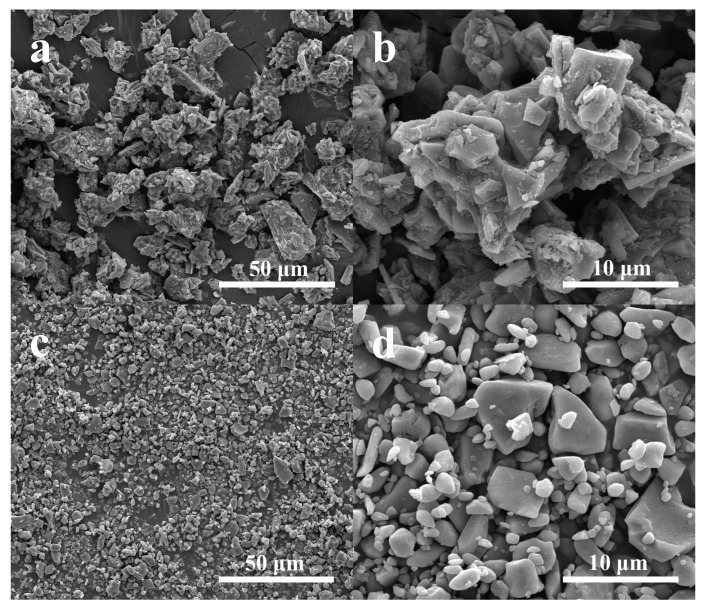
Scanning electron microscope (SEM) images of WSC/S (**a**,**b**) and NOSPC/S (**c**,**d**).

After the sulfur encapsulation process, the SEM images of WSC/S and NOSPC/S composites are displayed in Figure 2a–d, respectively. Compared with the original WSC, it is obvious that there are plenty of sulfur granules attached to the surface of WSC, which is mainly due to the lack of sufficient pores for WSC to accommodate active sulfur. Nevertheless, no aggregation of sulfur crystals is easily observed on the surface of NOSPC, which manifests the complete permeation of sulfur into the porous carbon substrate through a lateral capillary force in the melt-diffusion process [1]. Moreover, in contrast to NOSPC, some macropores/mesopores of the NOSPC/S are destroyed and micron-sized particles are acquired (approximately several to several ten micrometers), which could be caused by the grinding operation during the mixing process. To further survey the constituents of the NOSPC/S composite, the EDS mapping was implemented. As shown in Figure S2, the NOSPC/S composite contains multifarious elements including carbon, oxygen, nitrogen, and sulfur. We can see that oxygen and nitrogen were uniformly doped in the NOSPC, as well as that sulfur homogeneously infiltrated into the porous carbon framework.

**Figure 3:**
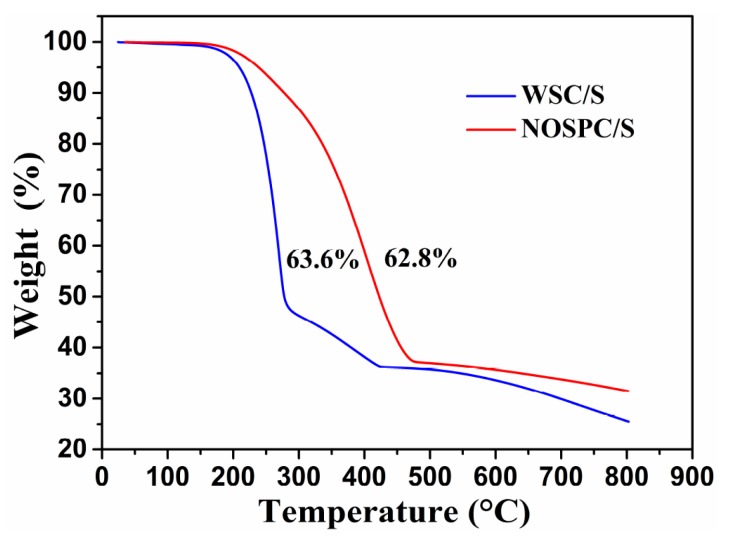
Thermogravimetric analyzer (TGA) profiles of WSC/S and NOSPC/S.

In order to quantify the sulfur amount in the WSC/S and NOSPC/S composites, TGA measurements were performed in an N_2_ atmosphere from indoor temperature to 800 °C. As displayed in Figure 3, both the composites began to lose weight at about 160 °C, and the curves reached stabilization when the composites were heated to exceeding 420 °C due to the sulfur evaporation. The sulfur contents in the WSC/S and NOSPC/S composites were 63.6 and 62.8 wt %, respectively, almost closing to the theoretical design sulfur contents. Furthermore, the TGA curve of the NOSPC/S sample exhibited a much higher evaporated temperature (470 °C) than that of the WSC/S composite (420 °C). This gives strong evidence that the NOSPC/S composite showed a stronger interaction between NOSPC and sulfur, which could be because of the retarding effect of the abundant porosity on the NOSPC framework [3,40].

**Figure 4:**
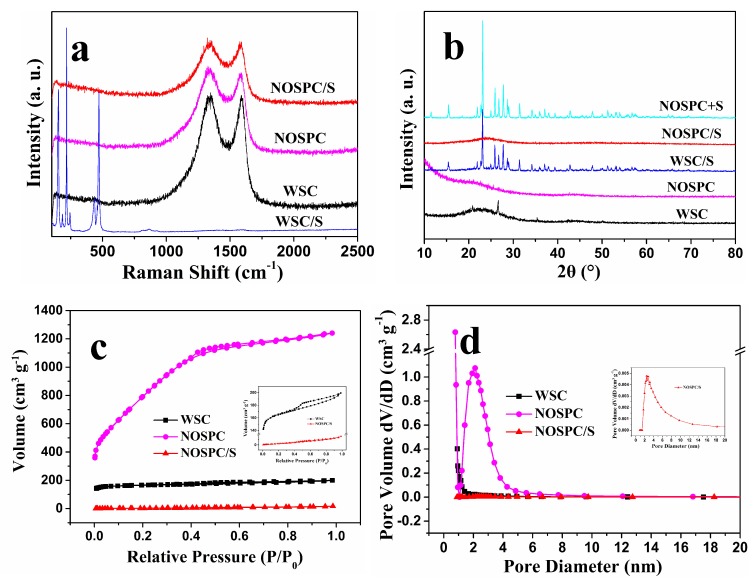
(**a**) Raman spectra of WSC, NOSPC, WSC/S, and NOSPC/S; (**b**) XRD curves of WSC, NOSPC, WSC/S, NOSPC/S, and the simple mixture of NOSPC/S powder; (**c**) N_2_ adsorption/desorption isotherms of WSC, NOSPC, and NOSPC/S; (**d**) pore size distribution curves of WSC, NOSPC, and NOSPC/S.

The Raman spectroscopy was also used to examine the structures of the WSC, NOSPC, WSC/S, and NOSPC/S, as shown in Figure 4a. It can be seen that Raman spectra of the WSC and NOSPC showed a representative graph of partially graphitized carbon with two bands at about 1330 cm^−1^ and 1590 cm^−1^ (D and G bands), respectively, which implies their good electrical conductivity. The D band usually represents disordered carbon structure associated with the edge sites, defects, and holes, while the G band is a typical character of crystalline graphite structure corresponding to sp^2^ bonding. Moreover, the relative intensity of D band (I_D_) and G band (I_G_) can indicate the disorder degree of carbon materials [40,41]. The Raman spectrum of NOSPC offered larger I_D_/I_G_ (1.08) than that of WSC (1.01), on account of the heteroatomic functional groups on the surface of NOSPC, as well as more defects and highly porous structure caused by KOH activation [41]. As for WSC/S and NOSPC/S, Raman spectroscopy of WSC/S shows a sequence of characteristic peaks below 500 cm^−1^ which can be assigned to the S-S bond [42], while that of NOSPC/S cannot detect any sulfur peaks. This phenomenon further confirms that sulfur is mainly covered on the WSC surface, while sulfur is successfully embedded into the pores of the NOSPC.

Figure 4b displays the XRD curves of WSC, NOSPC, WSC/S, NOSPC/S, and the straightforward mixture of NOSPC/S powder. The XRD spectrum of WSC and NOSPC show a wide specific diffraction peak between 20° and 30°, indicating that the synthesized WSC and NOSPC have a typical amorphous structure. The XRD pattern of the simple mixture of NOSPC/S powder normally exhibits a sequence of strong and sharp peaks with a well-defined Fddd orthorhombic structure of sulfur (JCPDS#: 08-0247) [7,43]. After sulfur infiltrating into the carbon substrate, the XRD pattern of WSC/S shows similar diffraction peaks to that of orthorhombic sulfur except their intensities become slightly weaker, manifesting that the majority of sulfur particles coat on the surface of WSC, which agrees well with the investigate in the SEM tests (Figure 2a,b). However, the XRD curve of NOSPC/S displays no characteristic diffraction peaks of sulfur, which means that elemental sulfur has triumphantly infiltrated into the pores of NOSPC and presented in a remarkably dispersed state [3], which is consistent with the SEM (Figure 2c,d) and EDS (Figure S2) analysis results.

The N_2_ adsorption/desorption isotherms derived from BET tests were obtained to quantitatively confirm the porous structure of WSC, NOSPC, and NOSPC/S. As illustrated in Figure 4c, the N_2_ adsorption/desorption isotherms for original WSC and NOSPC exhibit a high adsorption uptake in the relative pressure of P/P_0_ < 0.1, and a hysteresis loop within the scope of 0.5–0.99 P/P_0_, indicating that both the WSC and NOSPC are typically hybrid micro/mesoporous carbon. It is worth noting that the micropores of NOSPC are more luxuriant than those of WSC (Figure 4d and Figure S3). Moreover, the pore size distribution curve of NOSPC has a distinctive peak around 2.12 nm, indicative of the presence of partial mesopores, further strongly proving its hierarchical microporous/small- mesoporous structure. The apertures of NOSPC are mainly distributed in the area of less than 6 nm, this small size pore structure is reported to potentially limit the soluble polysulfides effectively through physical adsorption, thus decreasing the “polysulfides shuttle” and enhancing the sulfur utilization rate [44]. The specific surface area, total pore volumes, micropore volumes, and mean pore sizes of WSC, NOSPC, and NOSPC/S are then presented in Table 1. It was found that the specific surface area and total pore volume of WSC were 656.53 m^2^·g^−1^ and 0.309 cm^3^·g^−1^, respectively, while NOSPC had a higher specific surface area (3101.8 m^2^·g^−1^) and larger pore volume (1.92 cm^3^·g^−1^), which was caused by the KOH activation. In addition, the micropore volume of WSC (0.221 cm^3^·g^−1^) was also much less than that of NOSPC (1.457 cm^3^·g^−1^), which is consistent with the results of Figure 4d and Figure S3. The ultrahigh surface area of NOSPC favors the sulfur distribution in the carbon framework, the molten sulfur thus can easily permeate into the NOSPC by capillary force, the specific surface area and pore volume of the obtained NOSPC/S composites then decreased seriously (17.4 m^2^·g^−1^ and 0.026 cm^3^·g^−1^, respectively). Meanwhile, from Table 1 and the inset of Figure 4d, we can see that almost all micropores vanished and mesopores only partially reserved for NOSPC/S, which not only can endure the volume expansion during the charge/discharge process, and thus improve the cycling durability, but also can promote the full impregnation of electrolyte and the rapid transfer of lithium ions [2,16,45].

**Figure 5:**
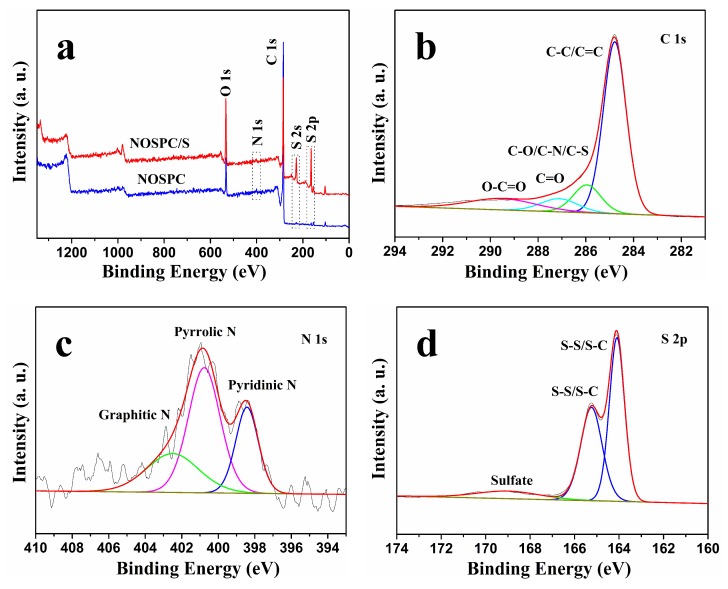
(**a**) XPS spectra of NOSPC and NOSPC/S; (**b**) high-resolution XPS spectrum of C 1s for NOSPC; (**c**) high-resolution XPS spectrum of N 1s for NOSPC; (**d**) high-resolution XPS spectrum of S 2p for NOSPC/S.

The XPS was utilized to analyze the chemical constituents and functional groups of NOSPC and NOSPC/S, and the corresponding outcomes were displayed in Figure 5. The XPS spectrum of NOSPC (Figure 5a) exhibited three obvious peaks corresponding to C 1s (284.8 eV), N 1s (400.9 eV), and O 1s (532.96 eV) electrons, as well as a minor peak related to S 2p (164.24 eV) electron, demonstrating the presence of N, O, and S heteroatoms. As calculated from the XPS results, the atomic percentages of C, O, N, and S in the NOSPC were 88.57, 7.43, 1.36, and 0.7 at %, respectively. The combustion elemental analysis of NOSPC also confirms the existence of C, O, N, and S elements (Table S1), which is consistent with the XPS measurements. However, the XPS spectrum of WSC (Figure S4) reveals the existence of C, O, and S elements, and the absence of N element. The difference in element compositions and contents between NOSPC and WSC may lead to the distinction of the electrochemical results when using as the sulfur substrates. As shown in Figure 5b, the C 1s XPS spectrum of NOSPC presents four prominent peaks at 284.8, 286.0, 287.1, and 289.5 eV, which correspond to C–C/C=C, C–O/C–N/C–S, C=O, and O–C=O, respectively [2,23,24], indicating that there were some oxygen-containing functional groups in the NOSPC matrix. The O 1s XPS spectrum of NOSPC also confirms the presence of oxygen functional groups (Figure S5). The high-resolution N 1s spectrum of NOSPC in Figure 5c presents a broad peak ranging from 396 to 407 eV. It can be fitted by three peaks allotted to pyridinic N (398.4 eV), pyrrolic N (400.8 eV), and graphitic N (402.5 eV), which is very common in the N-doped carbon materials [15,27]. The high-resolution S 2p spectrum of NOSPC can be deconvoluted into two peaks (Figure S6), which correspond to S–S/S–C bonds at 164.2 and 165.3 eV [4]. In addition, the survey spectrum of NOSPC/S (Figure 5a) obviously illustrates two classic peaks of sulfur (S 2s and S 2p), which are allotted to S_8_ [17]. In the S 2p XPS spectrum of NOSPC/S (Figure 5d), two fitted peaks situated at 164.1 and 165.2 eV are equivalent to S-S/S-C bonds. The wide peak at 169.0 eV corresponds to the sulfate, which may be caused by the oxygenation of elemental sulfur in the air [23,24]. The XPS test indicates that we have successfully prepared the N-, O-, and S-tri-doped porous carbon (NOSPC). Furthermore, these N/O/S- containing functional groups are believed to not only be able to improve the electronic conductivity of NOSPC [19,20,21], but can also further enhance the polysulfides anchoring capability via multidimensional chemisorptions, thus observably improving the cycling durability and rate capability of Li-S batteries [16,27,36].

**Figure 6:**
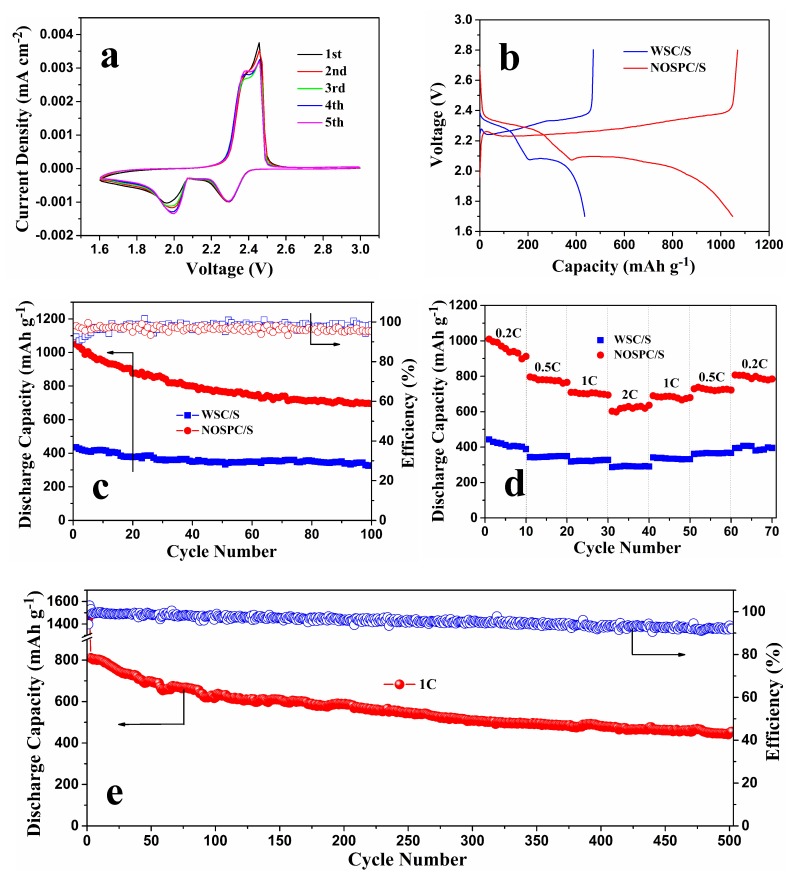
(**a**) CV curves of NOSPC/S at a scan rate of 0.2 mV·S^−1^; (**b**) the initial charge/discharge profiles of WSC/S and NOSPC/S at 0.2 C; (**c**) the cycling performances of WSC/S and NOSPC/S for 100 cycles at 0.2 C; (**d**) the rate performances of WSC/S and NOSPC/S at different current densities from 0.2 to 2 C; (**e**) the long cycling performance of NOSPC/S at 1 C.

The electrochemical properties of CR2025 coin cells assembled with lithium foils as the anodes and NOSPC/S and WSC/S composites as the cathodes were then studied. Figure 6a first displays the CV graphs of NOSPC/S at a sweep speed of 0.2 mV·S^−1^ within the voltage scope of 1.6–3.0 V. During the cathodic scan, there are two noteworthy reduction peaks roughly at 2.3 and 2.0 V, relating to the conversion of elemental sulfur to dissoluble lithium polysulfides (Li_2_S_x_, 4 ≤ x ≤ 8) and further from polysulfides to indissoluble Li_2_S_2_/Li_2_S, respectively [17,18]. In the succeeding anodic scan process, we can observe two partially overlapping oxidation peaks in the potential of approximately 2.3–2.4 and 2.4–2.5 V, which possibly corresponds to the transformation of Li_2_S_2_/Li_2_S to low-order lithium polysulfides and afterwards to high-order lithium polysulfides, respectively [7,46]. Remarkably, there was no evident difference in the redox peak currents and voltages in the next four cycles, confirming the high reactive invertibility and good electrochemical stability of the NOSPC/S composite electrode. In addition, it also indicated that the synergistic effects of NOSPC by structural restriction and multidimensional chemisorptions were extremely effectual in confining the diffusion of polysulfides and sustaining high active sulfur utilization in the redox reactions.

The initial galvanostatic charge/discharge profiles and cycling behaviors of the WSC/S and NOSPC/S composites at 0.2 C are presented in Figure 6b,c, respectively. Clearly, there are two classic voltage platforms roughly at 2.3 and 2.1 V, and only one broad voltage platform at 2.2–2.4 V in the both initial discharge/charge graphs, which is in much agreement with the outcomes of CV analyses (Figure 6a). Compared to the WSC/S cathode, the NOSPC/S cathode shows higher discharge plateau potential and lower charge plateau potential, that is, a smaller voltage gap, demonstrating a good invertibility and superior redox reaction kinetics of the NOSPC/S cathode [3,47]. The large electrode polarization of the WSC/S cathode may be caused by the insulative sulfur layer on the surface of WSC, which increases the resistance of the WSC/S electrode (Figure 2a,b). Furthermore, the initial discharge capacity of the NOSPC/S cathode at 0.2 C reaches up to 1049.2 mAh·g^−1^ (with a coulombic efficiency of 98.1%), contrasting to the 435.6 mAh·g^−1^ of the WSC/S cathode (with a coulombic efficiency of 92.5%). The higher active material utilization of the NOSPC/S cathode mainly results from the highly homogeneous decentralization of elemental sulfur in the pores of NOSPC, thus leading to a preferable electric contact between carbon and sulfur. At the same time, as illustrated in Figure 6c, the discharge capacity of the NOSPC/S cathode still remains 695.3 mAh·g^−1^ after 100 cycles at 0.2 C, while a pretty inferior discharge capacity of 326.3 mAh·g^−1^ is delivered for the WSC/S cathode. All in all, in comparison with the WSC/S cathode, the NOSPC/S cathode exhibits improved reversible capacity and cycling performance, which could be ascribed to the highly porous structure and in situ N-, O-, and S-tri-doping of NOSPC.

As shown in Figure 6d, the rate performances of the WSC/S and NOSPC/S cathodes were also investigated by augmenting the current density every 10 cycles from 0.2 to 2 C. Not surprisingly, the NOSPC/S cathode delivers much larger discharging specific capacities and superior rate capabilities at disparate current densities than the WSC/S cathode. After 10 cycles cycling at 0.2 C, the NOSPC/S cathode delivers the reversible capacities of 780.7, 700.5, and 619.2 mAh·g^−1^ at 0.5, 1.0, and 2 C, respectively. Importantly, a reversible capacity of 799.5 mAh·g^−1^ for the NOSPC/S cathode is recovered when the current is switched back to 0.2 C, indicating a favorable high-rate performance. Besides, Figure S7 displays the charge and discharge voltage graphs of WSC/S and NOSPC/S at different current densities from 0.2 to 2 C. We can see that both the WSC/S and NOSPC/S cathodes present the typical characteristic of two plateaus in the discharge and charge voltage graphs as the current densities increase gradually from 0.2 to 2 C. However, the charge and discharge voltage platforms of the NOSPC/S cathode are more obvious, and the corresponding voltage gaps are smaller, which further indicates that the NOSPC/S cathode has superior rate capability. The good rate capability of the NOSPC/S electrode mainly benefit from both high conductivity of the hierarchical porous structure and homogeneous distribution of N/O/S-containing functional groups, which synergistically expedite kinetic redox of polysulfides and repress shuttle effect of polysulfides.

The long-range cycling performance of the NOSPC/S cathode was also tested at a high rate of 1 C, as shown in Figure 6e. A discharge capacity of 810.1 mAh·g^−1^ was obtained at 1 C after the activation in the first two charged/discharged cycles at 0.05 C. Notably, the NOSPC/S cathode exhibited a distinguished cyclability by maintaining the high discharge capacities of about 585.9 mAh·g^−1^ after 200 cycles and 454.7 mAh·g^−1^ after 500 cycles at 1 C, respectively. Compared with the initial discharge capacity, the fading rate is only 0.088% over 500 cycles at 1 C, and the corresponding average coulombic efficiency is about 95.6%. Moreover, after 500 cycles, the decrease in coulombic efficiency is probably due to the consumption of a small amount of LiNO_3_ additive after prolonged cycling [48]. Besides, we have compared the electrochemical performances of NOSPC/S to some biomass-derived carbon materials in Li-S batteries (Table 2). We can see that the NOSPC/S cathode shows better cycle stabilities and rate capabilities when compared to previous reported biochar carbons [34,49,50,51]. The excellent electrochemical performances of the NOSPC/S electrode can be ascribed to the following merits of the NOSPC: (1) the conductive NOSPC can afford rapid electron transfer to accelerate the kinetic redox and thus enhance the sulfur utilization [52]; (2) the ultrahigh specific surface area and highly porous structure not only can ensure uniform dispersion of elemental sulfur and endure the volume expansion during the cycling, but can also promote the full impregnation of electrolyte to transfer the lithium ions rapidly and furnish enormous physical adsorption sites for polysulfides [1,2,6,35,45]; and (3) the in situ ternary N/O/S dopants in the NOSPC can further observably improve the conductivity of carbon materials and enhance the polysulfides-anchoring capability via multidimensional chemisorptions [16,27,36].

**Figure 7:**
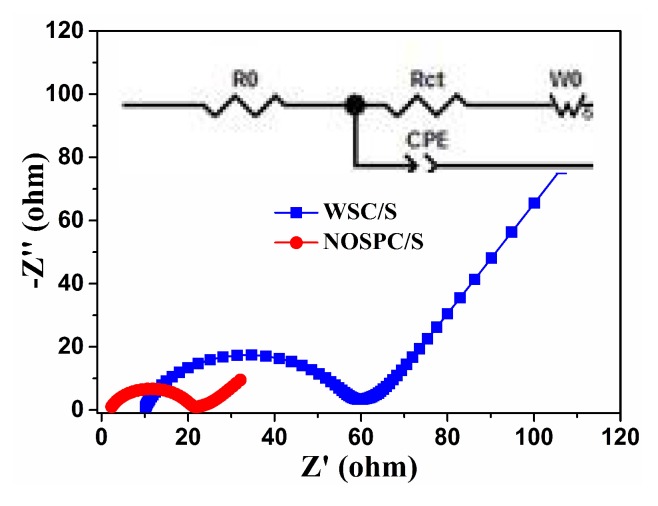
EIS curves of the fresh WSC/S and NOSPC/S electrodes, the inset is the relevant equivalent circuit model.

In order to further comprehend the interfacial charge transfer and ion diffusion process of the WSC/S and NOSPC/S electrodes, the EIS tests were implemented with button cells before discharge, as displayed in Figure 7 (the inset is the correlative equivalent circuit model). It can be perceived that both EIS curves are compose of a typical depressed semicircle at high frequency and a diagonal line in the low-frequency region. The intercept of the first semicircular in the solid axis Z’ is related to the combination impedance R_o_, which includes the interface impedance between active materials and current collector, the active materials’ intrinsic resistance, as well as the electrolytes’ ionic resistance [3,4]. The semicircle in the high-frequency region is the charge transfer resistance R_ct_, which corresponds to the kinetic resistance of electrochemical reaction at the electrode-electrolyte interface [53]. Depending on the correlative equivalent circuit model, we fitted the EIS curves and obtained the values of the corresponding impedances, as shown in Table 3. It can be seen that the R_o_ (1.88 Ω) value of the NOSPC/S electrode is much lower than that of the WSC/S electrode (9.82 Ω). It is because sulfur in the WSC/S electrode mainly exists on the surface of the WSC, while most of the sulfur permeates the pores of the NOSPC. Moreover, compared with the WSC/S electrode, the R_ct_ of the NOSPC/S electrode markedly decreases from 46.36 to 18.63 Ω. This is due to the conductive NOSPC can lower the resistance for electrons transport throughout the electrode, and the highly porous structure can facilitate ion transfer, thus leading to the decrease of R_ct_.

## 4. Conclusions

In summary, a novel N-, O-, and S-tri-doped porous carbon (NOSPC) has been triumphantly synthesized by pyrolyzing of natural wheat straw followed by KOH activation for the first time. Various test analyses demonstrate that the resulting NOSPC possesses a highly porous carbon framework, ultrahigh specific surface area, large pore volume, good electrical conductivity, and in situ N-, O-, and S-tri-doping. Due to the synergistic effects of physical confinement and multidimensional chemical adsorptions for repressing the polysulfides shuttle, the as-obtained NOSPC/S composite for the Li-S batteries delivers dramatically enhanced electrochemical properties including large initial discharge capacity (1049.2 mAh·g^−1^ at 0.2 C), good cycling stability (retains a reversible capacity of 454.7 mAh·g^−1^ over 500 cycles at 1 C with 0.088% capacity decay per cycle), and superior rate performance (619.2 mAh·g^−1^ at 2 C). This work will shed light on the reasonable design of carbon/sulfur composite materials with unique physical properties and surface chemistries, offering priceless guidance for the progress of advanced Li-S batteries for the practical applications.

## Figures and Tables

**Table 1:** The main pore parameters of WSC, NOSPC, and NOSPC/S.

Samples	Specific Surface Area (m^2^/g)	Total Pore Volume (cm^3^/g)	Micropore Volume (cm^3^/g)	Mean Pore Size (nm)
WSC	656.53	0.309	0.221	1.88
NOSPC	3101.8	1.92	1.457	2.48
NOSPC/S	17.40	0.026	0.00	5.95

**Table 2:** Electrochemical performances of Li-S batteries basing on different biomass-derived carbons.

Samples	S in the Cathode (%)	Rate (C)	Initial Capacity (mAh·g^−1^)	Cycle Capacity (mAh·g^−^^1^)	References
Wheat straw carbon	51.8	1	582	445(200th)	[34]
Chery pits carbon	45.6	1	550	410(200th)	[49]
Rice husk carbon	44.8	0.5	834	~600(200th)	[50]
Olive stones carbon	64	0.06	930	670(50th)	[51]
NOSPC	50.2	0.2	1049.2	766.9(50th)	This
		1	810.1	585.9(200th)	work

**Table 3:** The fitting results of the electrochemical impedance.

Samples	R_o_ (Ω)	R_ct_ (Ω)
WSC/S	9.82	46.36
NOSPC/S	1.88	18.63

## Supplementary Materials

The following are available online at http://www.mdpi.com/1996-1944/11/6/989/s1, Figure S1: SEM images of cross section (a,b), outside surface (c), and internal surface (d) for wheat straw, Figure S2: SEM image of NOSPC/S (a) and corresponding elemental maps of C (b), O (c), N (d), and S (e), Figure S3: Pore size distribution curve of WSC, Figure S4: XPS spectrum of WSC, Figure S5: High-resolution XPS spectrum of O 1s for NOSPC, Figure S6: High-resolution XPS spectrum of S 2p for NOSPC, Figure S7: Charge and discharge voltage profiles of WSC/S (a) and NOSPC/S (b) at different current densities from 0.2 to 2 C, Table S1: The combustion elemental analysis for NOSPC.
