# Supplementary material for: Wheat Straw-Derived N-, O-, and S-Tri-doped Porous Carbon with Ultrahigh Specific Surface Area for Lithium-Sulfur Batteries
Source: Materials (Basel). 2018 Jun 11;11(6):989. doi: 10.3390/ma11060989 (PMC6025088; doi:10.3390/ma11060989)
Supplement: Supplementary file 1 [file materials-11-00989-s001.pdf]

Materials **2018**, *11*, 989

materials

MDPI

Supplementary Files

Wheat straw-derived N-, O-, and S-tridoped porous carbon with ultrahigh specific surface area for lithium-sulfur batteries

Feng Chen [1], Lulu Ma [1], Jiangang Ren [1], Mou Zhang [1], Xinyu Luo [2], Bing Li [1], Zhiming Song [1,*] and Xiangyang Zhou [2,*]

[1] School of Resource and Environment, Henan University of Engineering, No. 1, Xianghe Road, Zhengzhou 451191, China; chenfeng871588@163.com (F.C.); malulu1001@163.com (L.M.); renjiangang2005@126.com (J.R.); mouzhang1015@163.com (M.Z.); hngclb@126.com (B.L.)

[2] School of Metallurgy and Environment, Central South University, Lushan South Street 932, Yuelu District, Changsha 410083, China; 17307484092@163.com

* Correspondence: songzhimin1961@126.com (Z.S.); hncsyjy308@163.com (X.Z.); Tel.: +86-0371-6250-8218 (Z.S.); +86-0731-8883-6329 (X.Z.)

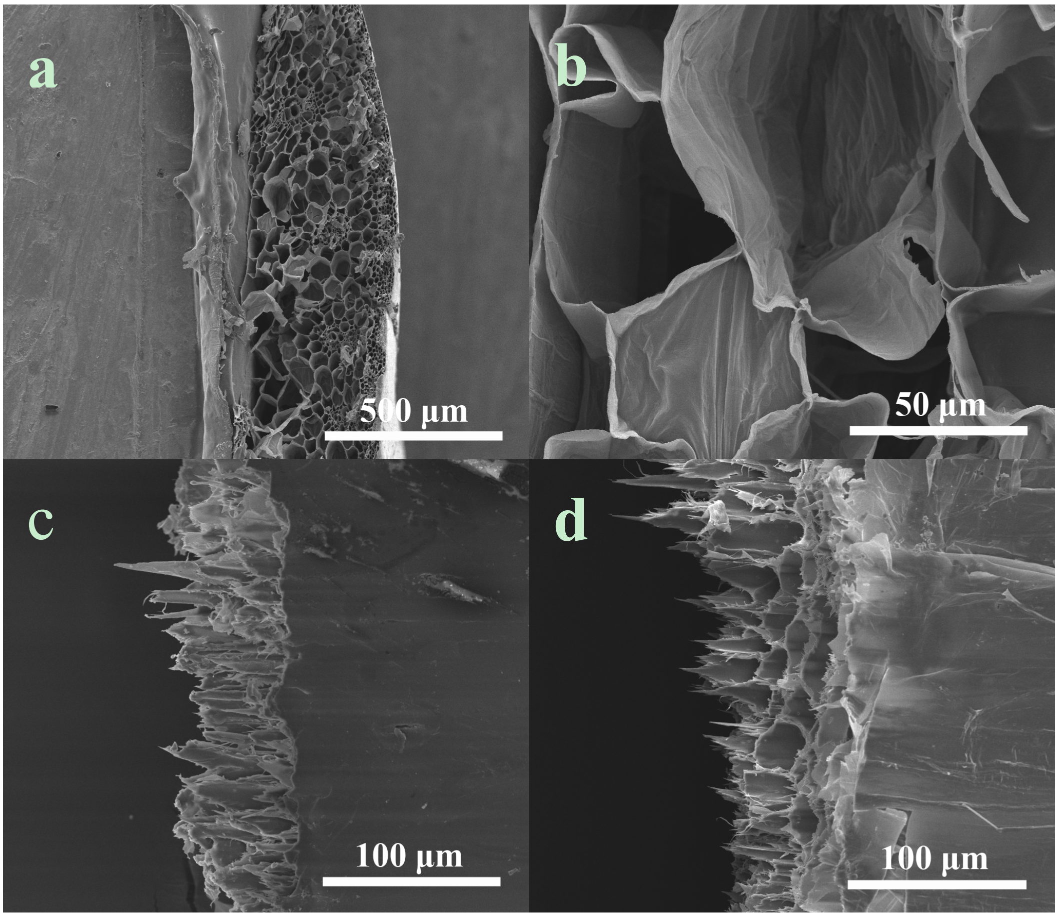

Figure S1. SEM images of cross section (a-b), outside surface (c), and internal surface (d) for wheat straw.

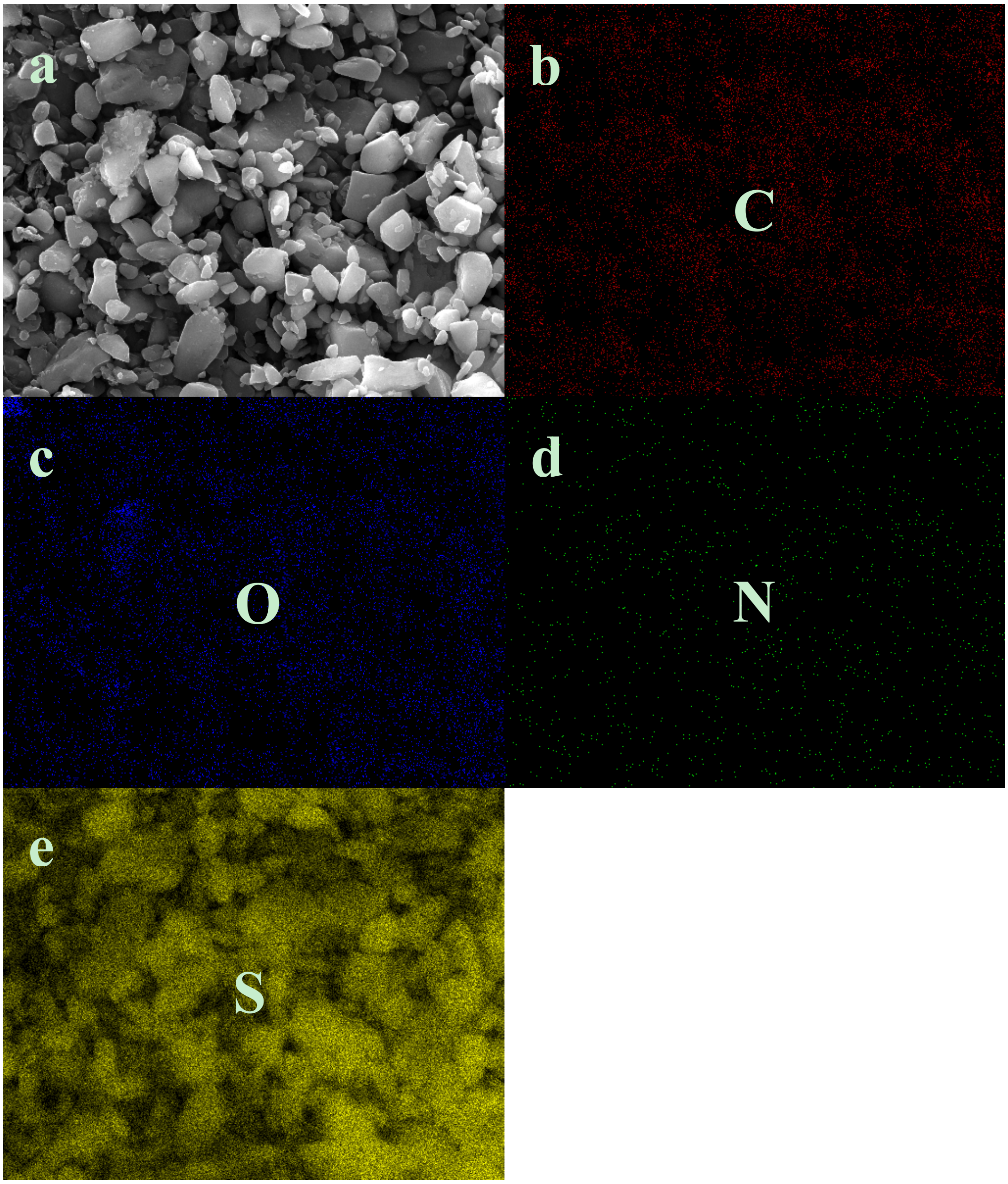

Figure S2. SEM image of NOSPC/S (a) and corresponding elemental maps of C (b), O (c), N (d), and S (e).

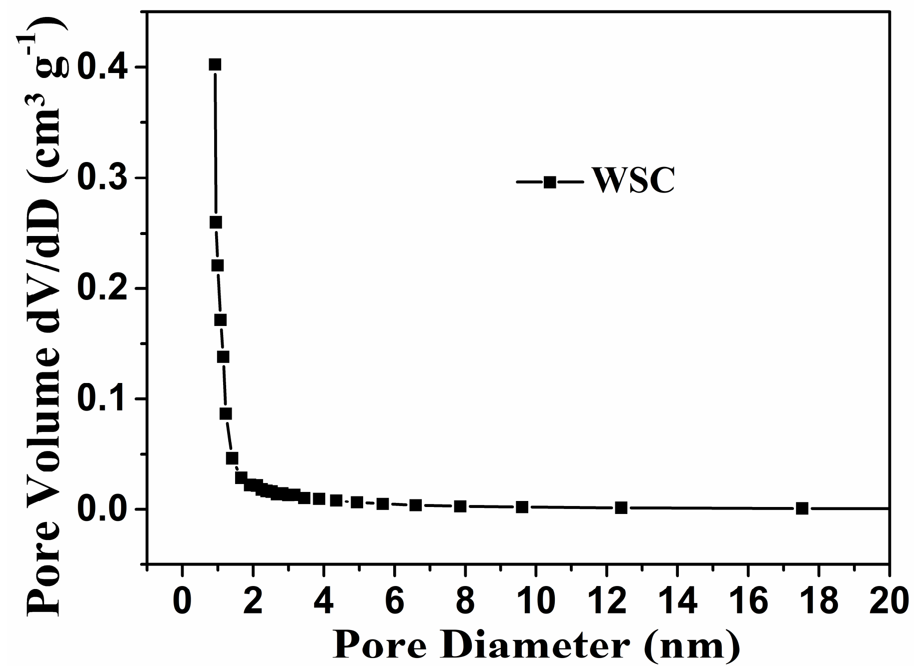

Figure S3. Pore size distribution curve of WSC.

Table S1. The combustion elemental analysis for NOSPC.

Samples	C [wt%]	H [wt%]	O [wt%]	N [wt%]	S [wt%]
NOSPC	80.59	0.18	9.12	1.01	1.35

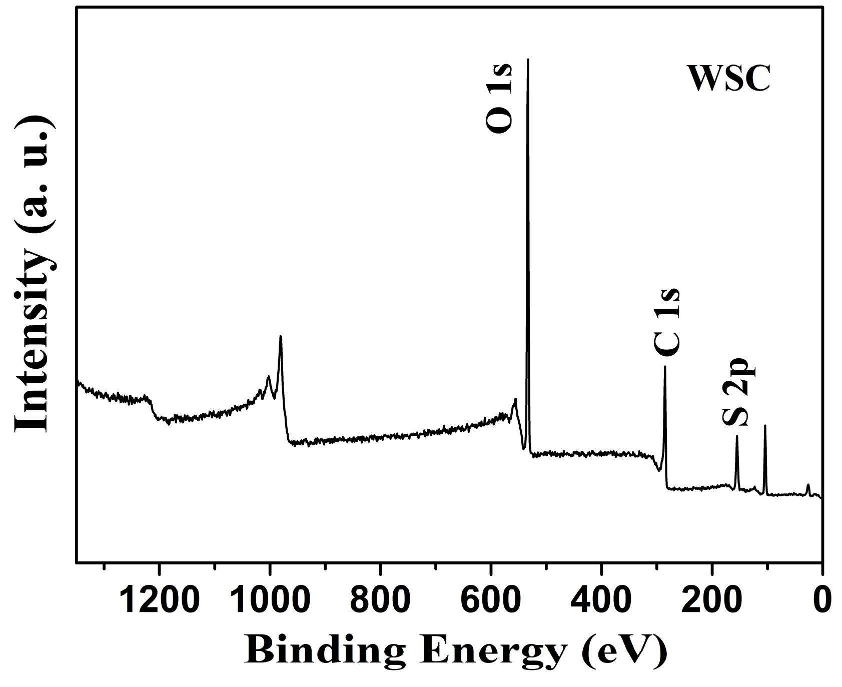

Figure S4. XPS spectrum of WSC.

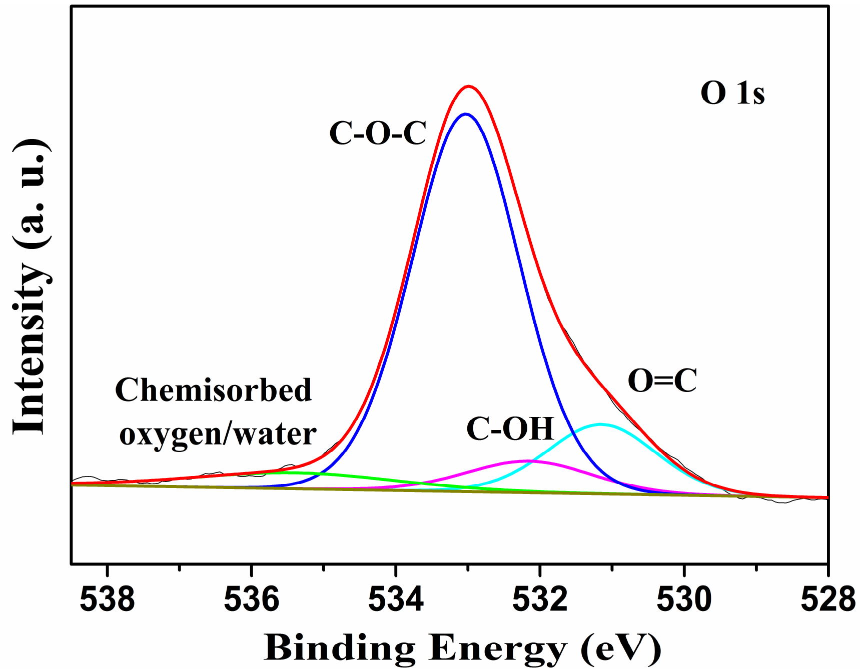

Figure S5. High-resolution XPS spectrum of O 1s for NOSPC.

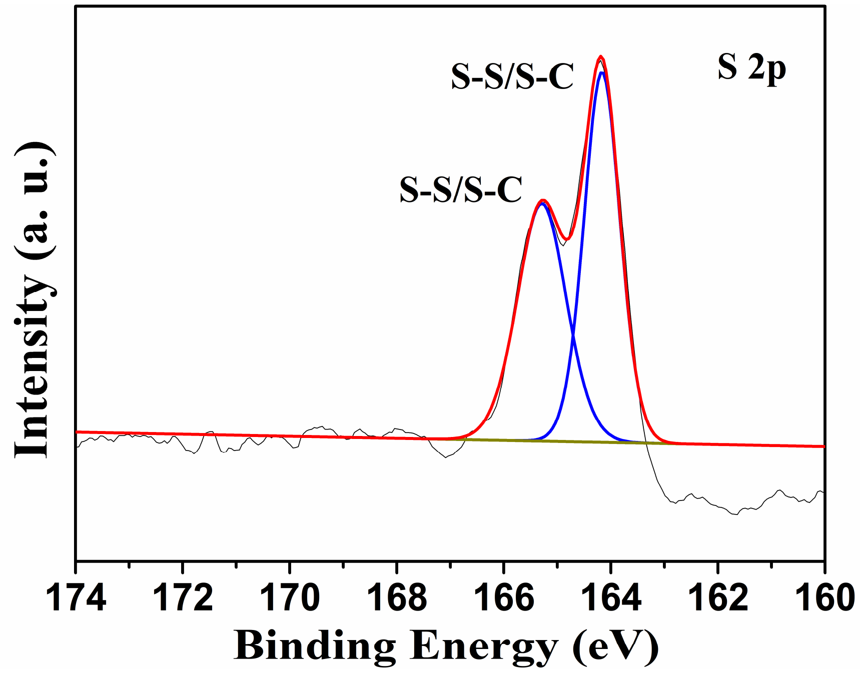

Figure S6. High-resolution XPS spectrum of S 2p for NOSPC.

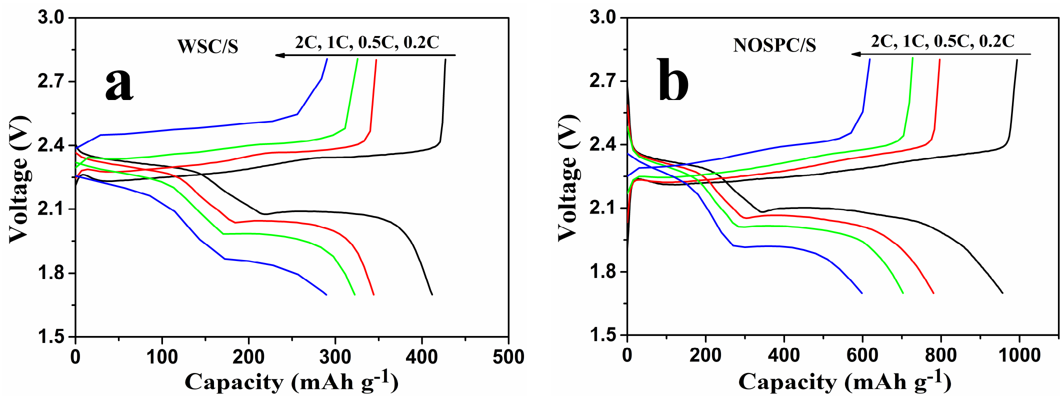

Figure S7. Charge and discharge voltage profiles of WSC/S (a) and NOSPC/S (b) at different current densities from 0.2 to 2 C.

© 2018 by the authors. Submitted for possible open access publication under the terms and conditions of the Creative Commons Attribution (CC BY) license (http://creativecommons.org/licenses/by/4.0/).
